# Pulmonary Administration: Strengthening the Value of Therapeutic Proximity

**DOI:** 10.3389/fmed.2020.00050

**Published:** 2020-02-27

**Authors:** Mafalda A. Videira, Jordi Llop, Carolina Sousa, Bruna Kreutzer, Unai Cossío, Ben Forbes, Isabel Vieira, Nuno Gil, Beatriz Silva-Lima

**Affiliations:** ^1^Departamento de Farmácia Galénica e de Tecnologia Farmacêutica, Faculdade de Farmácia da Universidade de Lisboa, iMed.ULisboa—Research Institute for Medicines and Pharmaceutical Sciences, Lisbon, Portugal; ^2^Radiochemistry and Nuclear Imaging Group, CIC biomaGUNE, Donostia-San Sebastián, Spain; ^3^Institute of Pharmaceutical Science, King's College London, London, United Kingdom; ^4^Champalimaud Centre for the Unknown, Lisbon, Portugal

**Keywords:** pulmonary administration, pre-clinical and clinical studies, inhaled nanomedicines, selective targeting, lung cancer, imaging, toxicity

## Abstract

In recent years inhaled systems have shown momentum as patient-personalized therapies emerge. A significant improvement in terms of therapeutic efficacy and/or reduction adverse systemic effects is anticipated from their use owing these systems regional accumulation. Nevertheless, whatever safety and efficacy evidence required for inhaled formulations regulatory approval, it still poses an additional hurdle to gaining market access. In contrast with the formal intravenous medicines approval, the narrower adoption of pulmonary administration might rely on discrepancies in pre-clinical and clinical data provided by the marketing authorization holder to the regulatory authorities. Evidences of a diverse and inconsistent regulatory framework led to concerns over toxicity issues and respiratory safety. However, an overall trend to support general concepts of good practices exists. Current regulatory guidelines^1^ that supports PK/PD (pharmacokinetics/pharmacodynamic) assessment seeks attention threatening those inhaled formulations set to be approved in the coming years. A more complex scenario arises from the attempt of implementing nanomedicines for pulmonary administration. Cutting-edge image techniques could play a key role in supporting diverse stages of clinical development facilitating this pharmaceutics take off and speed to patients. The ongoing challenge in adapting conventional regulatory frameworks has proven to be tremendously difficult in an environment where market entry relies on multiple collections of evidence. This paper intention is to remind us that an acceptable pre-clinical toxicological program could emerge from, but not only, an accurate and robust data imaging collection. It is our conviction that if implemented, inhaled nanomedicines might have impact in multiple severe conditions, such as lung cancer, by fulfilling the opportunity for developing tailored treatments while solving dose-related toxicity issues; the most limiting threat in conventional lung cancer clinical management.

## Introduction

Uncertainty over the conceivable safety issues over pulmonary administration has dramatically deemed its potential. Inhaled therapeutics or vaccines have the advantage of providing its accumulation in the target tissue avoiding its systemic spread and loss of pharmacological activity. Local drug accumulation with subsequent high local efficacy as it was demonstrated by the available data gathered from respiratory diseases, such as asthma, cystic fibrosis and chronic obstructive pulmonary disease (COPD) (1). Nanoparticle-based systems are cutting-edge feasible pulmonary nanomedicines, both for respiratory or non-respiratory diseases. Being developed as platforms they might represent for the pharmaceutical industry the much-needed flexibility to reach a pipeline of several rationales to provide inhaled formulations as a high-quality strategy against uncured diseases, such as, lung cancer.

Different indications of approved medicines reflect the need to fight multiple targets over cancer tumorigenesis, e.g., the complex microenvironment, growth rate, angiogenesis, dissemination and immunosuppressive properties. Concerning the clinical implications of innovative tools, in which drug “cocktails” and “sandwich-based” treatments have become more used, one challenge for the pharmaceutical technology is to fulfill unmet medical needs using more precise medicines.

Regulatory approval of breakthrough molecules, such as, immunotherapies, *nivolumab* (Opdivo®, Bristol-Myers Squibb), and *pembrolizumab* (Keytruda®, Merck) highlighted that pharmaceutical companies are still pursuing truly innovative solutions for lung cancer management. This trend can been also seen in the recent approved personalized therapies, such as *osimertinib* to be used in Epidermal Growth Factor Receptor (EGFR) exon 20 mutated lung carcinomas after failure of first line Tyrosine kinase inhibitors (TKI), or *crizotinib* (Xalkori, Pfizer) for first line metastatic ROS1 positive lung carcinomas, or even *alectini*b for anaplastic lymphoma kinase (ALK) positive carcinomas progressing after *crizotinib* approved for ROS1-positive metastatic non-small cell lung cancer (NSCLC). Up until today, limitations based on systemic toxicity, efficacy and safety of the available therapies along with the lukewarm effectiveness in including more selective, targeted delivery strategies, such as nanomedicines: broadly defined as drug delivery systems (nanoDDS) (2, 3).

Nanomedicines, were thought as a first-of-a-kind strategy designed to improve patient outcomes trough, (1) promoting selective deliver of their cargo in the lesion; (2) avoidance of adverse effects in healthy cells, and (3) protecting the pharmacological molecule from earlier metabolization (4). Typically, pharmaceutical innovation and strategy focus their efforts in designing carriers, such as liposomes, polymeric nanoparticles and micelles, specifically devised to improve the efficacy of loaded therapeutic agents by enhancing their bioavailability, stability and body residency upon intravenous administration (5–7). By contrast, there has been little effort to fight diseases, either locally or systemically, using inhaled therapeutics as a delivering system (8). Underexploring pulmonary administration of inhaled nanomedicines, their capability to reduce dosing and to fine-tune known drugs pharmacokinetics (PK), constitutes an unprecedented waste of a medical and economic competitive turning point.

Ever-increasing attention in nanomedicines, either marketed or under clinical trials, have already stressed that it is advisable to encourage a better understanding of this systems features by the regulatory authorities. Efforts already underway include task forces to find a consensus on the requirements for registration and market approval accelerating patient access to those products. Regardless the existing favorable “regulatory environment”^2^ and further reflection papers on inhaled formulations, the approval process remains heavyweight, unnecessarily time and cost consuming. High quality unbiased evidence on inhaled nanomedicines absorption, distribution, metabolization and elimination (ADME) should be generated based on robust experimental protocols and scientific guidelines. Otherwise, the existent constrains, and regulatory gaps might result in a redundant loss of understanding on how lung mechanistic and physiologic properties do affect drug pharmacology upon pulmonary administration.

Patients and physicians are looking forward to have the augured advanced inhaled nanoDDS pharmaceuticals and its consequent add value. However, despite their promising capabilities, differences in distribution, pharmacology, immunology and toxicology raise numerous issues needing to be addressed to hardness pre-clinical validation of new targets supported by original data collections.

Obstacles in the drug development process toward a broad acceptance of inhaled systems opportunities prompted several initiatives (9), such as the SimInhale working group hosted by the MP1404 COST Action^3^ By starting to recognize that pulmonary delivery systems and particularly inhaled pharmaceuticals, do represent a shift in drug conventional pharmacokinetics/pharmacodynamics (PK/PD), the challenge was to reassess experimental frameworks enforcing its utility to strengthen regulatory decision-making offices toward inhaled nanoDDSs approval, without losing the ethical safety-centered archetype neither the Quality Target Product Profile (QTPP) [ICH Q8] indispensable to provide safe drugs to patients fostering therefore future regulatory approval and output applications.

## Lung Cancer: Molecular Profiling Sets Sights for New Therapies

Embracing innovative solutions supported by biomedical data gathered from clinical/translational research studies could support clinical decisions that might contribute to improve lung cancer modest outcomes: primary cause of cancer-related death, with more than 1.6 million yearly deaths worldwide (10, 11). Nowadays, lung cancer patients face intensive and invasive treatment protocols that comprise surgery, chemotherapy, radiation therapy, or combinations thereof depending on: (1) the cancer type, (2) the stage of the disease, and (3) individual factors, such as, general health condition and lung function (12, 13).

In 2011, the International Association for the Study of Lung Cancer (IASLC), the American Thoracic Society (ATS), and the European Respiratory Society (ERS) established significant changes to the prior pathologic classification of lung cancer (11), with emphasis on correlations between pathologic aspects of tumors with clinical, radiologic, and molecular characteristic (14).

Conventional histologic-based classification for diagnosis and treatment decision in lung cancer have shown a limited scenario based in the dichotomist classification of small cell lung carcinoma (SCLC) and non-small cell lung carcinoma (NSCLC) (14–17). It lacks important information in terms of tumor “*omics*”^4^ thus narrowing the therapeutic possibilities and weakening the already complex rationale that underlies disease prognosis^5^.

### Precision Medicine for Genetically Defined Patients

Molecular-driven understanding of tumor growth and progression might positively impact areas, such as diagnosis and patient sub-populations outcomes. As metastatic competencies increase, several intracellular events drive the primary tumor communication with the surrounding microenvironment as well as with distant organs. At that point, it is possible to observe the emergence of complex molecular cascades (18, 19).

Systematic disease mutations in oncogenes crucial for sustaining cells tumorigenic potential, are perfect examples of conditions that could dictate patients stratification making possible to achieve outcomes beyond our current therapeutic expectations (20–22). Key signaling events involved in NSCLC growth, proliferation and tumor suppressive properties are related to the activation of specific oncogenes including through RAS/RAF/MEK/ERK and PI3K/AKT/mTOR axis (23, 24).

Clinical evidence gathered in patients with *EGFR* mutations or *ALK* rearrangements drove recent progresses in precision medicine field (25–30). This integrative methodology was used to support the approval of EGFR-TKIs (*gefitinib*, Iressa, AstraZeneca; *erlotinib*, Tarceva, Genentech/Astellas) as first-line treatment for patients with advanced NSCLC harboring somatic mutations in EGFR exons 19 and 21. Higher efficacy (progression-free survival (PFS) and safety (lower toxicity when compared with conventional chemotherapy), was reported in a phase III randomized clinical trials (16, 31).

Nanomedicines, mainly as inhaled nanoDDS, can be foreseen as a key “tool” for the implementation of personalized medicine. The concept of delivering selectively a therapeutic strategy to the lung region increasingly drives clinicians and researchers to address the cancer physiopathology as a cell mutation-based injury providing substantial enrichment in clinical disease management.

### Would Nanomedicines Restore the Cutting-Edge Expectations of Immunotherapies?

Disrupting tumor immunosuppressive mechanisms is already used by exploring different concepts: (1) Non-specific immune stimulation (32); (2) Adoptive cell transfer (33); (3) Vaccination strategies (34); and (4) immune checkpoint blockade (35, 36). Unsurprisingly, enhanced immune responses, though associated with better therapeutic outcomes and higher survival rates, might lead to severe toxicities. Nevertheless, the impact of immunotherapies on the induction and re-establishment of the immune system have been shaded mainly by the occurrence of irAEs (immune-related adverse events).

Lung cancers are characterized by the constitutive activation of oncogenes that regulate the abrogation of the normal T-cell's activation, driving tumor escape from the immune surveillance (36). These tumors signatures also rely in disturbance of immune checkpoints, e.g., CTL-4 and PD-1 or PD-L1 (37–40). Interfering with immune checkpoints through monoclonal antibodies (mAb) has been used successfully to amplify dendritic cell (DC)-derived immune responses (41–43).

Differing from the healthy conservative feedback mechanism, tumor's up-regulation of activated T-cells led to the overexpression of CTLA-4, generating a competitive unregulated arrangement (36, 37). Already approved *Ipilimumab*, a fully humanized anti-CTLA-4 mAb that targets protein receptors at the DC surface (44) is in a phase II trial as a first-line treatment of metastatic NSCLC, in association with chemotherapy. This combination displayed an improvement of the immune related progression-free survival in comparison with chemotherapy alone, without additional toxicity. *Tremelimumab* is another mAb directed against CTLA-4 that has been developed for several solid tumors, namely NSCLC (45).

Additional strategies against molecular targets have already progressed to clinical use, the first resulting from the pro-inflammatory cytokines released in the tumor microenvironment as well as the initial T-cell activation both up-regulating the membrane receptor PD-1, while the second is the PD-1 ligand (PD-L1), up-regulated by cytokines, such as INF-γ and IL-4, which are produced after T-cell activation, establishing a feedback loop that attenuates tumors immune responses (37, 38, 46–48). Anti PD-1 and PD-L1 therapies, such as *nivolumab* (Opdivo®), *pembrolizumab* (Keytruda®), *atezolizumab* (Tecentriq®), avelumab (Bavencio®), and *durvalumab* (Imfinzi®) are in clinical use for the treatment or co-treatment of a broad range of malignant stages. FDA-approval of *nivoluma*b (2014) has indications for patients with BRAF V600 wild type or mutation-positive unresectable metastatic melanoma. Lung cancer and specifically NSCLC patients already benefits from this therapy, as it becomes the first immunotherapy to be approved as second-line treatment (2015). Indications have now been extended to advanced renal, hepatocellular, and urothelial carcinomas, metastatic squamous cell carcinoma of the head and neck (SCCHN), classical Hodgkin's lymphoma (cHL) and microsatellite instability-high (MSI-H) or mismatch repair deficient (dMMR) metastatic colorectal cancer (49). Expectations in this molecular candidate have driven new clinical trials aiming: (1) investigate the efficacy of these mAb in combination with small targeted drugs/traditional chemotherapy; (2) to determine optimal administration patterns; and (3) to identify the subset of patients that may benefit the most from the new treatments (50, 51).

Recently, a combination of *ipilimumab* (anti-CTLA-4) and *nivolumab* (anti-PD-1) evaluated in a phase II trial against advanced melanoma, provided evidence of an improvement in patient response rate of 61%, compared to 11% for *ipilimumab* monotherapy, suggesting an exciting potential complementary role in regulating adaptive immunity (52, 53).

As broader utilization becomes a reality, more structured data identifies the strengths and weakness of its use. Unfortunately, from up to 90% of the patients treated with anti-CTLA-4, to 70% regarding to the anti-PD-1 or anti-PD-L1 therapy have experienced irAEs (54). Recent discouraging results have raised questions regarding the criteria to be followed for the selection of patients who may benefit from the use of anti-PD-1 monoclonal antibodies, e.g., patients with EGFR mutations or patients previously treated with TKIs (55, 56).

Lastly, despite some breakthrough innovations in the present clinical adopted protocols, the inclusion of conventional antitumor agents seems to remain mandatory. Together, the lack of cell selectivity and the known severe toxic side effects explain dose-limiting issues and consequent modest results in terms of survival rates. Adoption of precision medicines involves not only new molecules, supported by breakthrough knowledge in cell biology, but also their combination with novel administration strategies. Highly competitive therapeutic or diagnostic options could be foreseen by using pulmonary delivery of nanomedicines, either by addressing newly identified disease targets, thus solving unmet medical needs in lung cancer, or by decreasing conventional drugs administered doses (2, 3).

Though it is perhaps, unfair to claim that the actual classification of a tumor stage has been made over a conceptual weakness, the tremendously limited organ-based differentiation moves to a more precise mutation-driven disease classification.

Looking ahead, the sustainability of exploiting cell growth, signal transduction, angiogenesis, metastasis and cell cycle regulation as targets for new therapies regardless the cancer type may rely on nanoDDS transformative impact on the field. Bringing together new safety, quality and efficacy data, attracts the attention of regulatory authorities, and by doing so, genetically defined patients would greatly benefit from inhaled nanoDDS to delivery highly selective molecular-target agents or repurposing old, but indispensable, molecules.

## Nanomedicines in Pulmonary Administration

On top of the opportunities offered by nanoDDS as carriers for a broad range of therapeutic entities, it is now possible to capture some of the nanoparticle's features to achieve optimal pulmonary administration and radically change “drugs” bioavailability. Nonetheless, for aerodynamic engineered nanoparticles, tracking their biodistribution from initial particle deposition region through the alveolar and interstitial space to the drainage routes—pulmonary arteries, veins and lymphatic's is mandatory, together with identification of possible toxicological effects. Though the acquired knowledge regarding lung dynamics, namely the complex network responsible for the drainage of intrapulmonary fluids, also involved in cancer progression and metastases dissemination, emphasizes that no “simple approach” can be devised. Nevertheless, nanoDDS PK/PD, based upon the nanoparticle properties rather than the molecule's biochemical classification (BCC) can be design to improve lung distribution and deposition providing higher local pharmacological doses with attenuated systemic adverse effects (57, 58).

### Modulating Cell's Phenotype: RNAi-Based Therapy

Recent breakthroughs in the field of molecular biology and developments in RNA interference (RNAi) technology have promoted the therapeutic use of RNAi-mediated strategies in the oncologic field. Particularly, the use of short interfering RNA (siRNA) arose as a reliable therapeutic approach by which harmful genes can be “silenced” by delivering complementary and rationally designed sequences (59–62). Unlike conventional anti-cancer drugs (e.g., small molecules and antibody-based drugs), siRNA therapeutic potential is enormous due to unlimited possibilities in terms of targets and specificity, which are determined by the principle of complementary base pairing (63). Compare to other anti-sense alternatives, such as, DNA oligonucleotides, this approach is known to be safer, as it interferes at the post-translational level, avoiding mutation and teratogenicity risks (62, 64).

One of the major challenges in the siRNA field is the dependence of therapeutic efficacy on effective cell targeting. It is accepted that both cell uptake and intracellular trafficking are critical to promote intracellular accumulation of these molecular-target agents. Furthermore, limitations are also found in systemic administration, owing its quick degradation by body nucleases and clearance from the bloodstream. Unlike other molecules, siRNA biological fate is totally determined by the existence and properties of a carrier-mediated delivery system and its ability to overcome both extracellular and intracellular barriers (62).

Nanomedicines might promptly change the limitations already faced with intracellular delivery of naked oligonucleotides. The protection and selective targeting can undoubtedly generate opportunities for molecular-target agent's translation from bench to clinical use can be anticipated.

siRNA-nanocarriers engineered to tune selectivity of advanced nanoDDS liposomes, polymeric nanoparticles, micelles, solid lipid nanoparticles and inorganic nanoparticles could be designed in order to promote higher bioavailability, stability and residency times at the lung (65–67). For this reason, inhaled siRNA-based therapy deserves to be explored. The direct and localized administration of inhaled nano-entrapped siRNA might allow higher accumulation of siRNA in the tumor vicinity, being potentially the most valuable tool for the biomedical RNAi application in the near future (68). Newly identified cell targets and preliminary clinical findings with siRNA-nanocarriers reveal broad and diverse opportunities to impair tumor growth and dissemination (62).

Shim et al. (69) described the pulmonary delivery of cationic lipoplexes against myeloid cell leukemia sequence (siRNA, siMcl1). Different nanoliposomes exhibited similar behavior *in vitro* (in B16F10 cell lines), but variations *in vivo* (mouse). The nanoliposomes prepared with cationic dioleoyl-sn-glycero-3-ethylphosphocholine and cholesterol (ECL) presented the highest pulmonary delivery *in vivo* (26.2-fold higher than naked siRNA) and also the lowest cytotoxicity *in vitro* among the formulations tested. According to this study, the intra-tracheal administration of siMcl1 in ECL lipoplexes was able to significantly silence Mcl1 mRNA and its protein levels in the lungs, while reducing the formation of melanoma tumor nodules. These findings support the use of ECL nanoliposomes for pulmonary delivery of therapeutic siRNA to address lung cancer and other respiratory pathologies (69).

Currently Alnilam's cholesterol-modified siRNA for RSV treatment which is currently in Phase IIb and ExcellairTM (ZaBeCor, Bala Cynwyd, PA, USA), an inhaled siRNA-based treatment of asthma are the most used inhaled systems undergoing clinical trials (70).

### Adapting Pharmaceutical Dosage Forms to Achieve Pharmacological Levels Locally

Whichever the transported pharmacological agent, innovation, drug discovery and subsequent clinical trials have been mostly focused on intravenous administration, a route closely related to drug's adverse effects (71).

Exploring alternative administration routes, e.g., lung administration, are urgently required to offer a selective and more effective therapeutic delivery while preventing drug accumulation at non-targeted tissues. This innovative strategy also aims to reduce clinical adverse effects avoiding dose-limiting toxicities. Advances emerging from the use of nanomedicines are a major source of improvements that can be applied to explore this regional route of administration to handle lung diseases with emphasis in lung carcinomas (72, 73).

**Figure 1:**
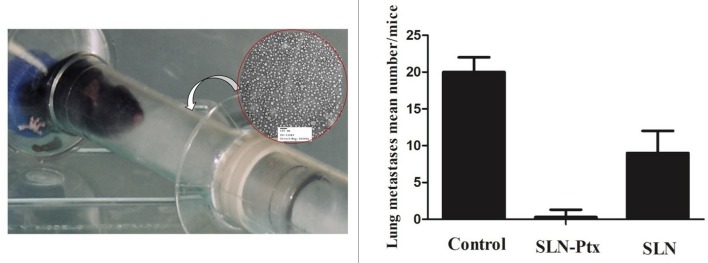
MXT C1.1 was selected due to its mesenchymal phenotype and its ability to produce lung metastases *in vivo*. Orthotopic implantation of cells (1 × 10^6^ cells) in the mammary fat pad of female BALB/c mice (Harlan Iberica, Barcelona, SP) was used to develop the primary tumor. Dissemination in distant organs, such as the pulmonary region was confirmed by histomorphology of the animal's lungs at the study endpoint (42 days). Animals were divided according to the inhaled formulation (randomized groups, *n* = 10): untreated control (group 1), plain SLN (group 2), paclitaxel-lipid nanoparticles (group 3). Each group received 6 administrations as depicted at the photo A mean tumor volume of 0.5 mm^2^ in the control group was considered the study “end point.” The primary tumors volumes were measured using a Vernier caliper. The results were expressed as the following: the prevalence of detected lung metastasis, as well as number and volume. The correlation between lung metastasis invasion with the paclitaxel-lipid nanoparticles inhalation was evaluated by regression analysis. *P*-values <0.05 were considered statistically significant.

In line with this observation, data from our research group shown activity against breast metastasis in lung, when paclitaxel entrapped in a lipid carrier system was administered *in vivo* by inhalation (74)^6^ Other experiments performed within our research team reinforced this inhaled nanoDDS efficacy against lung regional and distant metastasis *in vivo* upon transplantation of a spontaneous metastases model of the malignant breast cancer sub-population (MXT C1.1) (Figure 1). The entrapped drug (paclitaxel) proved to be more efficient when compared with the free drug administered I.V (74)^6^.

Tomoda et al. (75) reported a TAS-103 loaded PLGA-based nanomedicines against lung cancer, formulated in the form of a nanocomposite and administered by inhalation to rats. The authors found that the drug concentration in the lungs, following pulmonary administration, was significantly higher when compared to that obtained by I.V. of the free drug (75). Therapeutic evidences prove the potential of pulmonary administration of nanomedicines to the management of lung cancer or other cancer lung invasion (76).

## Non-Clinical Development of Inhaled Nanomedicines

### Nanoparticles PK/PD: Tracing the Systemic and Local Distribution

The effective transition of novel nanomedicines from experimental into the clinical arena demands a proper assessment of the properties forming the basis of their use (proof of concept plus kinetics plus toxicity). The relationship between the administered dose and the amount of drug deposited in the lung, the regional distribution, the residence time and/or clearance to the lung region or circulatory system need to be assessed for proper therapy planning (77).

For conventional drugs, this information is initially obtained in pre-clinical studies in animals and other test systems, using classical approaches, e.g., by analysis of biological samples (tissues, fluids) using a combination of techniques, such as high-performance liquid chromatography (HPLC) and mass spectrometry (MS) (78). In the case of nanomedicines, one of the main challenges arises from the fact that nanoparticles are extremely difficult to detect and quantify once distributed in an organism. Additionally, degradation or aggregation of the nanocarriers, interaction with biomolecules leading to alteration of the biological fate, and the process of drug release are extremely difficult to investigate using classical approaches (75, 79).

One strategy complementary to classical approaches for the tracking and quantification of nanoparticle-based complexes consists of using labeled particles, which implies the creation and use of dedicated technology for, (1) the labeling, (2) the visualization at organ, tissue, cellular and/or subcellular level of particles and drug-nanoparticle complexes, and (3) the quantification of nanoparticle-drug complexes, empty nanoparticles, non-complexed therapeutic molecule and degradation products (79, 80).

*In vivo, ex vivo*, and *in vitro* test systems are available or under development for the purposes above described, such as Positron Emission Tomography (PET) and Single Photon Emission Computerized Tomography (SPECT) (81). An excellent alternative to optical imaging techniques is the use of positron or gamma emitters. The disintegration of positron and gamma emitters leads ultimately to the generation of gamma rays (in the case of positron emitters, as the result of the annihilation of the emitted positron with an electron of a surrounding atom) which have virtually no penetration limits. Hence, if positron- or gamma emitter-radiolabeled nanomedicines are administered to experimental animals or eventually humans, the gamma rays resulting from the disintegration process can be externally detected and processed to generate 3D-images, which provide quantitative information about the spatiotemporal distribution of the labeled specie This is the basis of nuclear imaging techniques, i.e., PET and SPECT. When applying nuclear imaging techniques, appropriate selection of the labeling strategy is paramount to guarantee reliable results. The labeling strategy will ultimately depend on the physicochemical properties of the nanocarrier/drug, the selected radionuclide and the duration of the biological process to be investigated (81).

**Figure 2:**
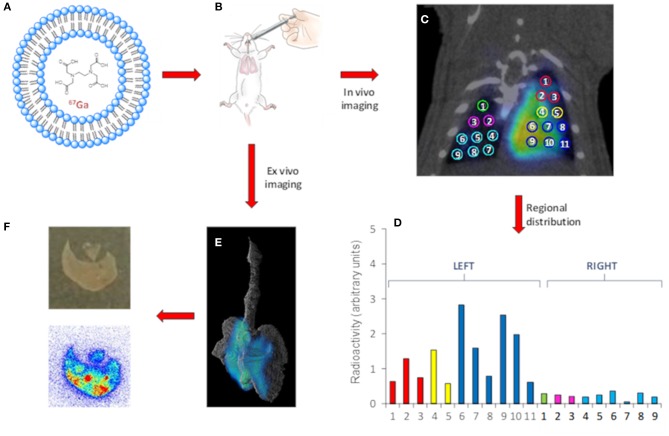
Representative workflow of the use of nuclear imaging to gain information related to the distribution of NCs after intratracheal insufflation. Gallium-67 labeled LPs **(A)** are administered to healthy rats. **(B)** *In vivo* **(C)** or *Ex vivo* **(E)** images can be obtained using SPECT. *In vivo* imaging enables quantification of the regional distribution. **(D)** *Ex vivo* autoradiography studies provide information about distribution at a higher spatial resolution **(F)** bottom. A photograph of the tissue slice is also shown **(F)** top. In the case shown, liposomes (LPs, as a model of nanocarrier) were labeled with Gallium-67 (gamma emitter, T1/2 = 3.3 days, E_max_ = 93, 184, and 296 keV) using a trans chelation method. The LPs were administered to healthy rats using the Penn-Century insufflators, and *In vivo* images were acquired using SPECT, enabling the determination of the regional distribution of the NCs within the lungs by drawing volumes of interest in the different lung lobes. In parallel studies, the lungs were harvested after appropriate tissue fixation and imaged using SPECT, resulting in more detailed images due to suppression of lung motion. Finally, autoradiography studies on tissue slices provided information about distribution at a higher spatial resolution.

Critical success factors such, as the accurate quantification *in vivo* owing the breathing-related motion of the subject during image acquisition. Of note, gated imaging can be applied to correct for this; alternatively, in the preclinical setting excision of the organs of interest and *ex vivo* imaging can solve this problem, at the cost of animal sacrifice. Additionally, resolution of nuclear imaging is usually poor (around 1 mm in the center of the field of view for preclinical systems, close to 5 mm for clinical systems). If higher resolution images are required, tissue dissection, slicing and evaluation using autoradiography can provide accurate information about regional distribution of the administered drug with a resolution close to 50 μm (Figure 2) (81).

Incorporation of labels both at the nanocarrier and the drug and subsequent tracking of the location of the different components integrating the nanomedicine can be envisaged, and information regarding biodistribution, residence time in the target organ or tissue and drug delivery kinetics *in vivo* might be achieved. This strategy, to the best of our knowledge, has so far not been applied to the investigation of drug loaded nanocarriers. However, recent works demonstrate the feasibility of the approach and hence energy-discriminant SPECT might become a powerful tool to gain information about the *in vivo* behavior of nanomedicines in the near future (80, 82).

### Safety Assessment

For inhaled drugs, bioavailability after pulmonary administration is a function not only of its characteristics, but also of the pharmaceutical formula and the physiological barriers that it must overcome. It has been postulated that the physiological deposition and adsorption mechanisms could be estimated based on the aerodynamic diameter of the inhaled agents. But this is a limited and controversy way to solve the problem and for the scientific way to solve the problem and for the scientific community does not take in consideration the lung histopathological state (83).

Few harmonized recommendations, such as the EMA/CHMP/QWP/49313/2005corr: Guideline on the Pharmaceutical Quality for Inhalation and Nasal Products; 3/26, have contributed for a harmonized environment mostly relative to chronic, combined chronic and carcinogenicity and carcinogenicity testing (84, 85).

The existing requirements for safety and efficacy evidence may not be adequate to describe nanoDDS' lung deposition and clearance. More important, conventional measure of AUC and C_max_ do not accurately predict the real clinical response upon pulmonary delivery, both at local and systemic level.

Given the different aspects, mainly the size and morphology, the evaluation of the nanosized-formulation impact on the safety attributes of the active agent shouldn't be focused on conventional pre-clinical tests alone. Up to now, functional pulmonary imaging technologies represent an important tool for the biodistribution assessment of nebulized nanoparticles.

## Pre-Clinical Safety Studies Aiming the Marketing Authorization for Pharmaceuticals

Regarding existing ICH guidance on the non-clinical requirements for support of clinical development and marketing authorization of medicinal products, mainly related to safety pharmacology the already existing guidelines (ICH 7a and I7b) and ICH topic M3 (2) lack aspects related to the lung specificity. This underlines the question: if the pre-clinical existing package seems to be insufficient for fostering the approval of the advanced inhaled pharmaceuticals what should be done to avoid this gap? This means a review on many aspects related to the value of non-clinical conventional safety evaluation (ICH Topic M3 (R2), biological issues associated with the active pharmaceutical ingredient bioavailability upon lung deposition but also considering critical the role of excipients, known and new, that are being used for preparing pulmonary delivery formulations.

Massive information has been gathered from research over the pulmonary administration route mechanisms and toxicological aspects. Not considering this fragment of evidence on emerging inhaled nanomedicines and their particularities represents a disadvantage for both industry and police makers.

Different situations may emerge in this exercise, for the development of medicinal products to administer by the inhalation route, (1) the repurposing of an existing nanomedicine, (2) the repurposing of an approved molecule using an innovative nanomedicine, (3) innovative molecule and formulation with well-characterized nanoparticles, and (4) innovative molecule and formulation with newly developed nanoparticles.

**Figure 3:**
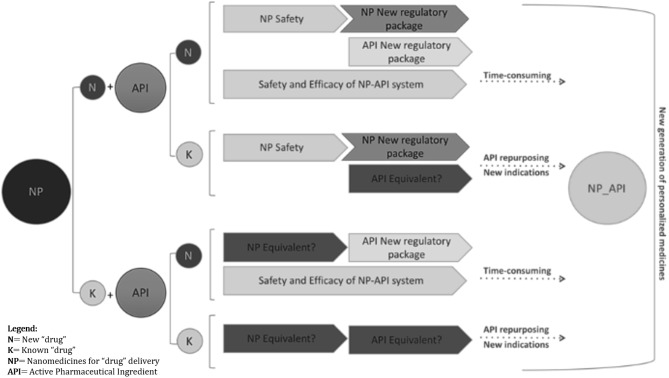
Different paths of regulatory requirements are summarized in the schematic diagram. Investigational new drug applications (IND), using existing or innovative nanoparticle formulations, will benefit from images supporting safety and efficacy evidence to complete “non-image” conventional end-points, pre-clinically and clinically, including quality and efficacy proof of concept, PK/PD, sub-acute to chronic toxicity, repeated dose toxicity, genotoxicity, carcinogenicity, reproductive toxicity, etc. Functional medical images of non-invasive lung administration either for inhaled active molecules or image agents can strengths the approval decision-make process. Bearing this in mind, regulatory acceptance of imaging-based evidence might have a time reduction impact in the benefit-risk assessment whenever image-guided “drug” delivery provides clinically significant data. Owing its potential to cover “drug” biodistribution cycle including body response and tissue alterations, and knowing that most of the new targetable cell events are common in cancer growth and survival, it is expectable that innovative molecules, targeting newly identified cell function alterations/abnormal signaling cascades, might have the decision-make process abbreviated even for broader indications.

Given the relatively large spectrum of proven or potentially useful molecular entities currently available, which show activity in lung cancer, or in non-lung tumors, but with plausible mode of action justifying their study in lung cancer, the hypothesis (1) and (2) might be the most straightforward for improving the tumor access of drugs through their local administration. For both concepts to be applied into nanomedicines development, multiple requests need to be fulfilled in what concerns the characterization of the biological effects and of the methodological tools used, e.g., the inhaled route and the delivery systems (Figure 3).

The clinical testing of such medicines demands a previous non-clinical estimation of the benefits (through proof of concepts) and of the risks posed by the new (pulmonary/inhaled) route of administration. Relevant methodologies are available, as described above, to address the distribution of the nanoDDS across the respiratory tract, their potential accumulation in the tumor vs. healthy cells, the intracellular distribution in relevant T-cells, and the biodistribution from the sites of administration and accumulation into the systemic circulation, with subsequent clearance. The comparison of these aspects within the delivery routes, e.g., the inhaled vs. the approved non-inhalation route (like I.V., or oral) will inform on the type and extension of studies that will be needed to support the entry into human trials.

Existing modeling and simulation approaches, together with tests in artificial respiratory tract/lung equipment's are becoming important tools for planning the particle characteristics vs. the intended deposition across the respiratory tract. If the systemic exposure, including tissue distribution of the inhaled formulation will be, as expected, lower than the observed with the approved molecule and formulation, the preclinical requirements will be mostly circumscribed to the local effects in the respiratory tract, meaning that a short-term repeated dose studies in one or two species might suffice to support the entry into human use.

On the contrary, depending on the comparative exposures reached systemically with the approved molecule vs. its innovative inhaled nanomedicines, a more extensive program will be needed, fully characterizing the (nano) particles, pre-clinical profile (e.g., ADME, long term toxicity, cellular fate) as well as the local toxicity.

This opens up countless opportunities for using the accumulated knowledge on existing molecules developed for non-lung tumors to support and abbreviate their development under inhaled formulations for lung cancer. The simplification of the development programs will allow faster patient access to innovation, but also will allow a faster success or failure together with increased knowledge on the relevance of the targeted cascades on the newly tested lung tumor(s), which are the remit of this manuscript. For now, it is important to focus our attention in toxicological matters rather than pharmacology and pharmacokinetics.

## Conclusions

The pulmonary route of administration embodies local/regional drug delivery against multiple diseases of the lung and the respiratory tract, e.g., asthma or cystic fibrosis. The systemic access of drugs through the lung has been attempted with limited success, as has been the case on inhaled insulin or calcitonin. Though this exploratory systemic administration shows great potential, information regarding possible advantages and challenges are out of this work scope.

The enormously potential value of the development of inhaled nanomedicines has been illustrated in this manuscript specifically for regional management of lung cancer or other cancers invasion in the lung compartment. This incurable disease may embody situations where a cost-benefit could be achieved by the local delivery of anticancer products. Furthermore, pulmonary administration followed by regional nanoDDS deposition would be expected to promote faster and more direct access of drugs to the tumor microenvironment, higher pharmacological concentrations even though with decreasing administered dosing and at the same time, reducing the systemic exposure and associated toxicities.

The increasing knowledge on the mechanisms behind tumors environment and the associated cellular/functional cascades, provides evidences on shared mechanisms not exclusive of one tumor type, making possible to approve therapeutic strategies with indications for multiple tumors in different organs. In another words, tumor characterization based on identified mutations rather than lesions in specific tissues/organs open up the rationale for shared therapies among multiple cancer situations independent of their location.

Numerous challenges need to be overcome for a successful and efficient pulmonary delivery of antitumor products, including the “construction” of appropriate formulations based on nanoparticles having appropriated quality attributes for reaching and targeting the intended cell, within the “geography” of the respiratory system, taking into consideration the physiopathological changes inherent to the disease itself.

Furthermore, any innovative formulation aiming to deliver new or existing therapeutic molecules will need to undergo a pre-clinical and clinical development program for supporting the marketing authorization stage and further human administration. Pre-clinical programs will include proof of concept, PK/PD and toxicology studies, being the extension of the studies determined by the innovative components of the developing medicines.

Medicines repurposing will be a consequence of these new approaches, which mat allow a faster application of innovation to multiple situations. The preclinical programs may therefore be reduced and adjusted when the repurposing of a drug is attempted for changing the target organ of efficacy. A faster and better patient access to innovative treatments will be a direct beneficial consequence. These considerations apply to pulmonary delivery of existing medicines approved for use through other administration routes or intended for different organ targeting.

Tracking nanoDDS across the pulmonary tract, different lung cells and eventually subcellular systems, demands technical solutions that are emerging, such as imaging technologies. Nevertheless, it is expectable that one of the higher advantages of local pulmonary delivery of antitumor drugs, particularly for lung cancer will be the reduction of the doses needed and reduction of systemic toxicity.

The early and continuous dialogue between the scientists involved in the development process and regulators will be fundamental for the design of well-structured, potentially more simplified development programs, pre-clinical and/or clinical, to allow an earlier access and benefit of patients to those innovations.

## Author Contributions

MV, JL, and CS managed the writing/manuscript preparation and data presentation. MV and JL were also responsible for the funding acquisition. BK, IV, BF, and BS-L conducted and prepared the manuscript section about toxicity and regulatory issues. NG provided a critical review on the clinical status and UC conducted the experimental work.

### Conflict of Interest

The authors declare that the research was conducted in the absence of any commercial or financial relationships that could be construed as a potential conflict of interest.

## Footnotes

^2^The European Medicines Agency's scientific guidelines on nanomedicines help medicine developers prepare marketing authorization applications for human medicines.

^3^COST (CO-operation in Science and Technology). Pan-European intergovernmental framework dedicated to European-based S&T networking activities under the EU Framework Program Horizon 2020. https://www.cost.eu/who-we-are/about-cost/.

^4^Genomics, proteomics, transcriptomics, etc.

^5^NSCLCs (80–85% of lung cancer) were grouped together disregarding more specific histologic subtypes (i.e., adenocarcinoma, squamous cell carcinoma, and large cell carcinoma), though no differences in the therapeutic systemic approach where considered for each subtype.

^6^Dissertation thesis is available in *Repositório da Universidade de Lisboa* in the following URL: http://hdl.handle.net/10451/271.
